# Overexpression of TWIST2 correlates with poor prognosis in Head and Neck Squamous Cell Carcinomas

**DOI:** 10.18632/oncotarget.390

**Published:** 2011-12-22

**Authors:** Daniela Gasparotto, Jerry Polesel, Alessandra Marzotto, Roberta Colladel, Sara Piccinin, Piergiorgio Modena, Alessandra Grizzo, Sandro Sulfaro, Diego Serraino, Luigi Barzan, Claudio Doglioni, Roberta Maestro

**Affiliations:** ^1^ Unit of Experimental Oncology 1, CRO National Cancer Institute, Aviano, Italy; ^2^ Unit of Epidemiology and Biostatistics, CRO National Cancer Institute, Aviano, Italy; ^3^ Unit of Pathology, Pordenone City Hospital, Pordenone, Italy; ^4^ Unit of Otorhinolaryngology, Pordenone City Hospital, Pordenone, Italy; ^5^ Unit of Pathology, San Raffaele Institute, Milano, Italy

**Keywords:** TWIST1, TWIST2, SNAI1, SNAI2, EMT, HNSCC

## Abstract

Head and neck squamous cell carcinomas (HNSCC) are a heterogeneous group of tumors with variable presentation and clinical behavior. Despite improvements in surgical and radiation therapy techniques, the 5-year survival rate has not improved significantly over the past decades. Thus, there is an urgent need to identify novel markers that may allow for the development of personalized therapeutic approaches. In the present study we evaluated the prognostic role of the expression of genes related to the induction of epithelial mesenchymal transition (EMT). To this aim, a consecutive series of 69 HNSCC were analyzed for the expression of TWIST1, TWIST2, SNAI1, SNAI2, E-Cadherin, N-Cadherin and Vimentin.

TWIST1, TWIST2, SNAI1 and SNAI2 were significantly overexpressed in HNSCC, with TWIST2, SNAI1 and SNAI2 being more markedly increased in tumors compared to normal mucosae. The expression of TWIST1 and SNAI2 was associated with upregulation of mesenchymal markers, but failed to correlate with pathological parameters or clinical behaviour. In contrast, we found that upregulation of TWIST2, which was independent of the activation of a mesenchymal differentiation program, correlated with poor differentiation grade (p=0.016) and shorter survival (p=0.025), and identifies a subset of node-positive oral cavity/pharynx cancer patients with very poor prognosis (p<0.001).

Overall our study suggests that the assessment of TWIST2 expression might help to stratify HNSCC patients for risk of disease progression, pointing to TWIST2 as a potential prognostic marker.

## INTRODUCTION

Head and neck squamous cell carcinomas (HNSCCs), which include malignancies originating from oral cavity, pharynx and larynx, are a heterogeneous group of tumors with variable presentation and biological behavior. The estimated worldwide incidence is about 500,000 new cases per year [1], with over 63,000 events/year in Europe [2].

Although HNSCC is highly curable when diagnosed at early stage, the majority of patients present at the diagnosis with loco-regionally advanced disease (stage III-IV), which results in poor prognosis. In fact, despite improvements in surgical and radiation therapies, still HNSCC remains a cancer associated with a high mortality rate. The recent integration of systemic chemotherapeutic therapy has slightly improved survival but the toxic effects are not negligible [3-5]. Unfortunately, conventional staging parameters, such as site and size of the tumor, lymph node involvement or presence of distant metastases (TNM), fail to provide a clear-cut risk stratification, which prevents the development of personalized approaches. Thus, there is an urgent need for the identification of new and more reliable diagnostic/prognostic markers and the validation of novel therapeutic targets.

Recent studies indicate that the phenomenon of epithelial-mesenchymal transition (EMT) plays a key role in carcinoma aggressiveness. EMT is a process whereby a cell loses the epithelial constraints of cell-cell and cell-extracellular matrix interactions, undergoes cytoskeleton reorganization and gains a mesenchymal phenotype. This is accompanied by a reprogramming of the transcriptional machinery that results in *de novo* activation of mesenchymal markers, such as N-Cadherin and Vimentin, and loss of epithelial intercellular adhesion molecules, such as E-Cadherin [6]. Essential for the development of embryonic mesoderm [7, 8], EMT is potentially destructive if deregulated, and it is becoming increasingly clear that inappropriate utilization of EMT mechanisms is an integral component of malignant progression of several epithelial tumors [9]. Two classes of transcription factors, including the TWIST proteins TWIST1 and TWIST2 and the SNAI proteins SNAI1 and SNAI2 play a pivotal role in the induction of EMT. Neo activation of these genes, that are essentially silent in normal epithelial tissues, has been reported to correlate with EMT in several types of cancer, including breast, colon, stomach, thyroid, and hepatocellular carcinomas [10-17]. Aberrant expression of these transcription factors has been associated also with the bypass of oncogene-induced failsafe programs (apoptosis and premature senescence) and drug resistance, suggesting that TWIST and SNAIL proteins may impinge upon pathways that are both dependent and independent of transdifferentiation [18-26].

Based on this body of information, we sought to investigate the role of EMT molecules TWIST1, TWIST2, SNAI1 and SNAI2 in HNSCC development and assess their potential diagnostic/prognostic value.

## RESULTS

### Gene expression

Demographic, clinical and pathological features of the 69 HNSCC cases analyzed in this study are shown in Table 1.

**Table 1:** Demographic and clinical-pathological characteristics of HNSCC patients

Characteristic		n	(%)
**Gender**	Male	65	94.2
	Female	4	5.8
**Age**	<60	41	59.4
	≥60	28	40.6
**Anatomic site**	Tongue	17	24.6
	Oral cavity	3	4.3
	Oropharynx	13	18.8
	Hypopharynx	20	29.0
	Larynx	16	23.2
**pT status**	1	6	8.7
	2	15	21.7
	3	20	29.0
	4	28	40.6
**pN status**	0	20	29.0
	1	5	7.2
	2b	18	26.1
	2c	23	33.3
	3	2	2.9
	X (unknown)	1	1.4
**Histological grading**	Well differentiated	2	2.9
	Moderately differentiated	37	53.6
	Poorly differentiated	30	43.5
**Stage**	I	4	5.8
	II	5	7.2
	III	10	14.5
	IV	50	72.5

**Figure 1:**
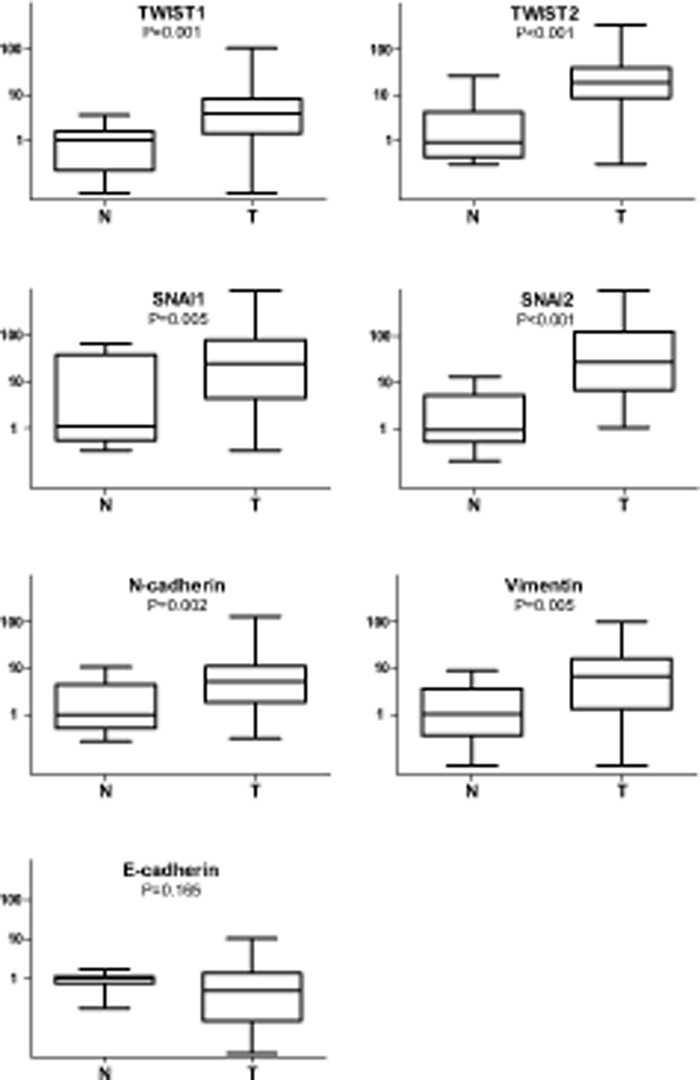
Box-and-whiskers plot representing relative expression levels of EMT-related genes in HNSCC (T) and normal mucosae (N) Gene expression levels are shown on the ordinate (log scale). The upper and lower horizontal lines of the box correspond to the first and third quartiles, the middle horizontal line the median, the whiskers maximum and minimum values. Wilcoxon-Mann-Whitney test was used to compare median values in normal and tumor samples.

Expression levels of the mRNA encoding TWIST1, TWIST2, SNAI1, SNAI2, N-Cadherin and Vimentin were all significantly increased in tumors compared to healthy mucosa, with TWIST2, SNAI1 and SNAI2 displaying the greatest increments (Figure 1). In particular, the extent of the increase, calculated as the ratio of the median value detected in tumors vs the median value detected in normal mucosae, was 3.8 fold for TWIST1, 20.9 for TWIST2, 23.4 for SNAI1, 29.5 for SNAI2, 5.2 for N-Cadherin and 6.3 fold for Vimentin. A trend toward reduction of E-cadherin mRNA expression in tumors compared to normal tissues could be observed, although it was not statistically significant.

**Figure 2:**
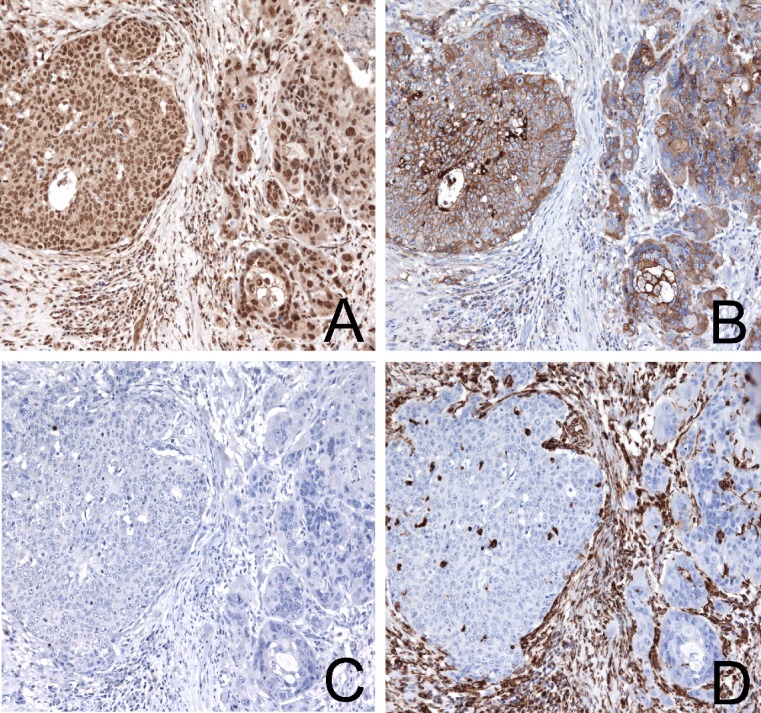
Immunohistochemical staining of a hypopharyngeal squamous cell carcinoma with high TWIST2 expression not associated to EMT The tumor displays a strong TWIST2 reactivity (A) accompanied by E-cadherin expression (B) and is negative for Vimentin (C) and N-cadherin (D). Magnification 200x.

EMT is conventionally described as a coordinate activation of mesenchymal markers (e.g. Vimentin and N-cadherin) and loss of expression of epithelial markers (e.g. E-cadherin). The activation of either TWIST1, TWIST2, SNAI1, SNAI2 resulted in a negligible downregulation of E-cadherin, while TWIST1 and SNAI2 displayed a good correlation with the expression of Vimentin and N-cadherin (Table 2). These latter were also positively correlated one to the other. In contrast, SNAI1 and TWIST2, which were reciprocally correlated, were independent of the activation of mesenchymal markers (Vimentin and N-cadherin) (Table 2). The lack of correlation between TWIST2 and induction of a canonical EMT phenotype was confirmed also at the protein level (Figure 2).

**Table 2:** Expression of EMT-associated genes in HNSCC: Pearson's correlation coefficients (all tumor sites)

	E-Cadherin	N-Cadherin	Vimentin	SNAI1	SNAI2	TWIST1	TWIST2
**E-Cadherin**	1	0	0	-0.1	-0.1	-0.1	-0.1
**N-Cadherin**		1	0.7	0.1	0.3	0.5	-0.1
**Vimentin**			1	0.1	0.7	0.6	0.1
**SNAI1**				1	0.1	0.1	0.6
**SNAI2**					1	0.5	0.1
**TWIST1**						1	0
**TWIST2**							1

**0.2 < r < 0.3 weak association 0.3 ≤ r < 0.5 moderate association r ≥ 0.5 strong association**

To better assess the role of these genes in HNSCC and to corroborate our results, a systematic analysis of 11 gene expression datasets available in Oncomine and in EBI Array Express databases was carried out. The various pro-EMT transcription factors (TWIST1, TWIST2, SNAI1 and SNAI2) displayed different degrees of reciprocal interdependence in different datasets. In particular, in most datasets TWIST1 and SNAI2 were reciprocally correlated, as well as the expression of Vimentin and N-cadherin, which is in agreement with our data. Moreover, the co-upregulation of these mesenchymal markers (Pearson's correlation coefficient r > 0.2) was associated to a significant downregulation of E-cadherin (r < -0.2) in only 3 out of 11 datasets (Supplementary Table 1), indicating that de novo gain of mesenchymal features may coexist with the expression of epithelial markers in a relevant proportion of HNSCC. The analysis supported also the role of TWIST1 and SNAI2 in the induction of an EMT program in HNSCC. In fact, the overexpression of Vimentin and N-cadherin was associated to the upregulation of TWIST1 in 7 out of 11 datasets and of SNAI2 in 6 out of 11 datasets. Similar to what observed in our series, TWIST2 expression failed to correlate with Vimentin and N-cadherin in the majority of datasets including this probe set, corroborating our observation of a marginal role for this TWIST family member in the induction of EMT in HNSCC.

Having collected evidence that, different from TWIST1, TWIST2 does not correlate with gain of mesenchymal features, we sought to corroborate this observation by in vitro experiments. To this end, we ectopically expressed TWIST1 and TWIST2 in FADU cells, that are reported to be susceptible to EMT [27]. Different from TWIST1, ectopic expression of TWIST2 failed to result in significant transcriptional modulation of EMT markers, thus supporting the concept that TWIST2 is not involved in the promotion of EMT in the context of HNSCC (Supplementary Figure 1).

### Pathological correlations

No significant associations between TWIST1, TWIST2, SNAI1, SNAI2, E-Cadherin, N-Cadherin or Vimentin and patient's age, gender, tumor site, size, nodal status or staging were found (Supplementary Table 2). Instead, a positive correlation between TWIST2 expression and histopathological differentiation grade was detected: TWIST2 was significantly increased in poorly differentiated HNSCC, as compared to well/moderately differentiated tumors (p=0.02, Wilcoxon-Mann-Whitney test). The positive association between TWIST2 and histopathological grading was also confirmed by categorization of tumors into high and low gene expressors, considering the median as a cutoff value (p=0.016, Fisher exact test, Supplementary Table 2).

### Clinical correlations

As a preliminary step to the evaluation of the role of EMT in patient survival, we first assessed how conventional clinical-pathological parameters performed in our tumor series. In agreement with the literature, survival rates at 5 years where higher for larynx compared to oral cavity/pharynx cancer patients (tongue, oral cavity, oropharynx, hypopharynx). Moreover, positive nodal status, advanced tumor stage, and poor histopathological differentiation grade correlated with worse prognosis (Supplementary Table 3).

**Figure 3:**
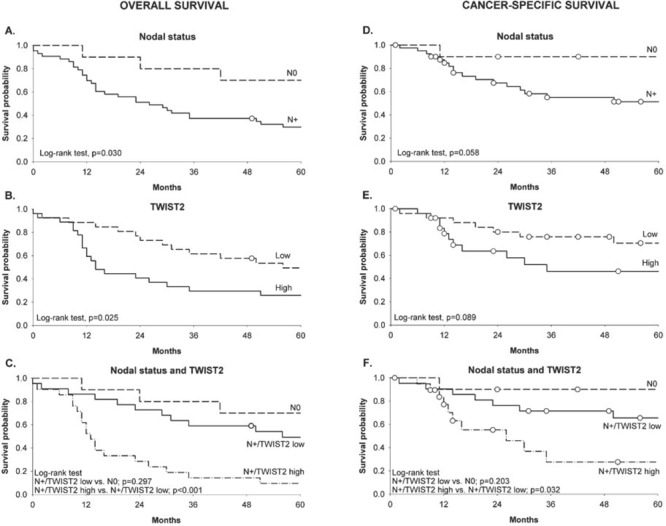
Kaplan-Meier estimates of overall survival (left panel) and cancer-specific survival (right panel) in oral cavity/pharynx cancer patients according to nodal status (A, D), TWIST2 expression (B, E) and combined nodal status and TWIST2 expression (C, F) White dots represent censored subjects.

To assess the prognostic value of EMT-related genes, tumors were categorized according to their relative gene expression levels into high and low expressors, using the median as a cutoff value. We found that high expression levels of TWIST2 significantly correlated with shorter survival in oral cavity/pharynx cancer patients, even after adjustment for tumor grading (Table 3 and Figure 3). No significant correlation was observed for larynx cancers but the limited number of node-positive laryngeal neoplasms included in our series (5 cases) prevented us from drawing any definitive conclusion about the significance of TWIST2 in this tumor context (Supplementary Table 3). More interestingly, TWIST2 demonstrated a remarkable prognostic value when combined with patient's nodal status. In fact, node-positive tumors overexpressing TWIST2 (node-positive/TWIST2-high) displayed a significant poorer outcome compared to node-positive tumors expressing low levels of the gene (node-positive/TWIST2-low) (Figure 3). Thus, TWIST2 expression identifies a group of node-positive oral cavity/pharynx cancer patients highly prone to progression.

**Table 3:** Kaplan-Meier estimates, hazard ratio of death (HR) and corresponding 95% confidence intervals (CI) according to gene expression levels in oral cavity/pharynx tumors

			Overall Survival		
**Gene**	Expression levels§	n	5-years survival probability	Log-rank test	HR (95% CI)^†^
**TWIST1**	Low	27	29%		1^‡^
	High	26	46%	p=0.168	0.62 (0.31-1.24)
**TWIST2**	Low	26	49%		1
	High	27	26%	p=0.025	2.18 (1.08-4.41)
**SNAI1**	Low	27	49%		1
	High	26	26%	p=0.519	1.32 (0.64-2.72)
**SNAI2**	Low	27	33%		1
	High	25	44%	p=0.306	0.71 (0.35-1.44)
**E-Cadherin**	Low	29	34%		1
	High	23	39%	p=0.882	1.06 (0.53-2.13)
**N-Cadherin**	Low	27	37%		1
	High	22	41%	p=0.878	0.95 (0.46-1.98)
**Vimentin**	Low	26	34%		1
	High	26	42%	p=0.541	0.83 (0.41-1.68)

§ Expression levels equal/below (Low) or over (High) the median value.

† Estimated by means of Cox hazard.

‡ Reference category.

Of the 11 datasets previously analyzed, only the dataset by Chung and coworkers included both TWIST2 probesets and cancer-specific survival data (dataset XI, Supplementary Table 1) [28]. Similar to our result, also in Chung's dataset high expression levels of TWIST2 correlated with shorter survival, although, possibly because of the limited size of the series (28 cases), the differences failed to reach statistical significance (Supplementary Figure 2).

## DISCUSSION

The much unexplained variability in the biological behaviour and clinical course is one of the major factors that hamper implementation of optimised risk-adjusted therapeutic strategies for HNSCC [3, 4]. Recent studies indicate that the activation of an EMT program in carcinomas may significantly contribute to tumor progression [9-16]. On this basis we sought to investigate the role of EMT-related molecules in HNSCC development and aggressiveness.

Our study provides evidence that both the EMT transcription factors (TWIST1, TWIST2, SNAI1 and SNAI2) and EMT markers (Vimentin and N-cadherin) are upregulated in a significant fraction of HNSCC, as compared to normal mucosa. TWIST1 and SNAI2 were reciprocally correlated, which is in line with recent evidence indicating that SNAI2 is a transcriptional target for TWIST1 [29]. Moreover, the expression of TWIST1 and SNAI2 was positively correlated with the co-activation of Vimentin and N-cadherin, in agreement with the reported role of EMT drivers for these transcription factors [9, 12, 14]. Overall, these data suggest that the initiation of a mesenchymal transition program involves a significant fraction of HNSCC.

A systematic analysis of a series of 11 publicly available transcriptional profiling HNSCC datasets corroborated the concept that TWIST1 and SNAI2 are likely key regulators of EMT in these tumors. In fact, in the majority of the datasets analyzed the expression of either TWIST1 or SNAI2 was associated with the co-activation of Vimentin and N-cadherin. In these data sets the co-upregulation of these mesenchymal markers did not necessarily associate with loss of E-cadherin expression, which is in line with our results and with recent findings by Nguyen and coworkers [30]. This suggests that in HNSCC the activation of a mesenchymal switch may be at least in part uncoupled from transcriptional downregulation of E-cadherin. Analogous observation has been recently reported also for non-small cell lung cancer [31].

Although the expression of TWIST1 and SNAI2 correlated with the co-activation of Vimentin and N-cadherin and hence with the initiation of a mesenchymal differentiation program, in our tumor series they failed to correlate significantly with clinical-pathological characteristics. A positive correlation between TWIST protein expression and poor prognosis in HNSCC has been recently suggested by Ou and coworkers [32]. However, the antibody used by these authors recognizes both members of the TWIST family, which prevented the actual assessment of the specific role of TWIST1 and TWIST2 in this phenomenon. We provide evidence that both genes are involved in HNSCC, but TWIST2 is more markedly overexpressed compared to TWIST1 (median fold-increase: 20.9 for TWIST2, 3.8 for TWIST1). More importantly, we demonstrate that TWIST2 correlates with high tumor grade and short survival in oral cavity/pharynx cancer patients and identifies, within the N-positive category, a subset with high risk of progression. A correlation between TWIST2 expression levels and worse prognosis has been recently suggested for tongue [33] and salivary adenoid cystic carcinomas [34] and a similar trend could be observed also in the HNSCC series analyzed by Chung et al. [28].

Intriguingly, the expression of TWIST2 failed to correlate with the activation of a mesenchymal transition program (Vimentin/N-Cadherin upregulation and E-cadherin downregulation), as confirmed both at transcriptional and protein level. This suggests that TWIST2 likely contributes to HNSCC aggressiveness via a mechanism different from the simple reprogramming of differentiation. In particular, the association of TWIST2 expression with grading and survival might reflect the ability of this gene to inhibit cellular safeguard programs, promote “stemness” and affect inflammatory response. In fact, TWIST2 has been reported to participate to stem cell renewal [35-38], to antagonize stress-induced apoptosis and to prevent oncogene-induced failsafe programs by interfering with both p53 and RB pathways [18, 20, 23, 39]. Moreover, elevated protein levels have been associated to aberrant proliferation and chromosome instability [21, 40] and, accordingly, we found that TWIST2 is overexpressed in high grade tumors that are characterized by elevated mitotic index, nuclear pleomorphisms and atypical mitoses. Finally, TWIST2 has been shown to negatively modulate the NF-kB-mediated production of pro-inflammatory cytokines and therefore to affect the immune response [41, 42]. Thus, TWIST2 could contribute to the poor prognosis of HNSCC by impinging on different pathways.

It should be pointed out that there is likely an overall underestimation of the role of TWIST2 in cancer. This is in part due to the fact that only very recently TWIST2-specific antibodies have been made available and in part to the limited number of transcriptional arrays including TWIST2-specific probes. In fact, TWIST2 gene has been only recently properly annotated and TWIST2-specific probes were included in commercially available arrays only since 2004 (Affymetrix U133 Plus 2.0).

Overall our data indicate that the gain of mesenchymal features seems to impact marginally on the clinical behavior of HNSCC. Rather, our study suggests that the assessment of TWIST2 expression, particularly in node-positive oral cavity/pharynx tumors, might help in identifying those cases that are at higher risk of fatal progression, pointing to TWIST2 as a potential marker. Although our study was performed on a limited number of cases, we consider that our results are worthy of further investigations and, if confirmed in a larger series, may provide novel criteria for risk stratification and disclose original therapeutic avenues for HNSCC patients.

## MATERIALS AND METHODS

### Tumors and patients

Sixty-nine patients with primary HNSCC undergoing surgery at the Unit of Otorhinolaryngology of the Pordenone City Hospital (North-East Italy) were enrolled in this study, prior approval of the institutional review board. No patient had received chemotherapy or radiotherapy treatment prior to surgery. Samples, readily collected after surgical resection, were histologically inspected by the pathologist, dissected with a scalpel blade, snap-frozen in liquid nitrogen and then stored at –80°C until use. Before RNA extraction a section representative of the frozen sample was evaluated under the microscope to ascertain the high representation in tumor cells. As a control, 14 samples of matching healthy mucosa, dissected free of non mucosal tissues, were also evaluated. Demographic, clinical and pathological features are shown in Table 1. Most patients were males (65 males, 4 females) with loco-regionally advanced disease (stage III–IV). Tumors included 53 oral cavity/pharynx tumors (17 tongue, ICD-9 141; 3 oral cavity, ICD-9 144; 13 oropharynx, ICD-9 146; 20 hypopharynx, ICD-9 148), and 16 larynx tumors (ICD-9 161). Histopathological classification and staging were based on the WHO classification and on UICC/TNM system [43]. Information on survival was obtained through an active follow-up based on verification of vital status of patients. Median follow up was 45 months.

### Purification of RNA and c-DNA synthesis

Frozen tissues were dismembered with a TissueLyser (Retsch). Total RNA was extracted by the EZ1 RNA universal tissue kit on the EZ1 biorobot (QIAGEN) and treated with DNAse to eliminate DNA contamination. c-DNA was synthesized starting from 1 microgram of RNA by the Superscript III reverse transcriptase (Invitrogen), according to manufacturer's protocol.

### Quantitative real-time PCR

The relative expression of TWIST1, TWIST2, SNAI1, SNAI2, E-Cadherin, N-Cadherin, Vimentin was quantified by real-time RT-PCR the using the SYBR® green PCR master mix and the ABI7900HT Sequence Detection System (Applied Biosystems). Transcript levels were normalized against GAPDH using the standard curve method for quantification, and expressed in arbitrary units relatively to a calibrator. For each gene, relative expression was obtained by dividing expression values by the median of normal samples. All results are means of three technical replicates. All data were confirmed on at least two independent RT-PCR reactions and, in 12 cases, also on two independent RNA extractions.

The following primers were used: TWIST1 (Ref. Seq. NG_008114.1), TWIST1-F: TCCTCTACCAGGTCCTCCA; TWIST1-R: GGAAACAATGACATCTAGGTCTC; TWIST2 (Ref. Seq. NM_057179.1), TWIST2-F: AGCGACGAGATGGACAATAAGATGACC; TWIST2-R: CGGTCCGGAGGTGGGTGGCG; E-Cadherin (CDH1) (Ref. Seq. NM_004360), CDH1-F: AAGGAGGCGGAGAAGAGGAC CDH1-R: CGTCGTTACGAGTCACTTCAGG; N-Cadherin (CDH2) (Ref. Seq. NM_001792.3), CDH2-F: GGTGGAGGAGAAGACCAG, CDH2R-R: GGCATCAGGCTCCACAGT; SNAI2 (Ref. Seq. NM_003068.3), SNAI2-F: ACATTAGAACTCACACGGGGA; SNAI2-R: GTGTGCTACACAGCAGCCAGA; SNAI1 (Ref. Seq. NM_005985.2), SNAI1-F: CTGCAGGACTCTAATCCAGAGTT SNAI1-R: CGGTGGGGTTGAGGATCT; Vimentin (VIM), (Ref. Seq. NM_003380.3) VIM-F: ACAACCTGGCCGAGGACATC, VIM-R: AGAGACGCATTGTCAACATCCTG; glyceraldehyde-3-phosphate dehydrogenase (GAPDH), (Ref. Seq. NM_002046.3) GAPDH-F: CGGGAAGCTTGTGATCAATGG, GAPDH-R: GGCAGTGATGGCATGGACTG.

PCR conditions were as follows: 95°C for 10 min, 95°C for 15 sec, 55-63°C for 1 min for 40 cycles.

### Immunohistochemistry

Five μm sections were immunostained with an anti-Twist2 polyclonal serum (AF 6249, R&D systems) which was first validated for absence of cross-reactivity against Twist1 on ectopically expressed human Twist1 and Twist2. Sections were also stained for E-cadherin (clone 36B5,Novocastra, 1:100), N-cadherin (clone IAR06, Novocastra, 1:400) and Vimentin (clone V9, Dako, 1:400). Staining procedures were performed by an automated immunostainer (Bond System, Leica Microsystems) using a non-biotin detection system (Bond Polymer Refine, Leica Microsystems) and diaminobenzidine development. Heat-induced antigen retrieval was performed using Tris-EDTA buffer (pH 9.0) in a water bath at 95°C for 30 min.

### Transfections

FADU, a cell lines derived from a squamous cell carcinoma of the hypopharynx, was purchased from ATCC. Cells were transfected by the calcium-phosphate method with either myc-tagged TWIST1, TWIST2 or control pCDNA3 empty vector. Seventy-two hours following transfections, cells were harvested and analyzed by western blot for protein expression using the following antibodies: mouse monoclonal anti-myc tag (clone 9E10, Calbiochem); anti-Twist1 (clone 2C1a, Santacruz), anti-E-cadherin (36/E-Cadherin, BD Biosciences), anti-N-cadherin (clone 32, BD Biosciences), anti-GAPDH (clone 6C5, Millipore) and rabbit polyclonal antibody anti-Twist2 (AF 6249, R&D Systems).

### Statistical analysis

Wilcoxon-Mann-Whitney and Fisher's exact tests were used to compare gene expression levels in tumors and normal mucosae and to investigate the association with clinical-pathological parameters. Pearson's correlation coefficient was used to assess the correlation between gene expression levels. The prognostic value of EMT-related genes was studied by dividing the tumors into two groups, high-expressors and low-expressors, using the median value of each gene analyzed as a cutoff. The effect of individual factors on overall survival was compared through hazard ratios (HR) and corresponding 95% confidence interval (CI), estimated by means of Cox proportional hazard model. Survival analysis was computed by Kaplan-Meier method, and log-rank test was used to evaluate the differences between subgroups. Statistical significance was claimed for values ≤ 0.05 (two-sided).

### Analysis of publicly available transcriptional profiles

In order to assess the role of EMT drivers in HNSCC, a series of 11 independent datasets, deposited in the Oncomine (www.oncomine.com) or in the EBI Array express database (www.ebi.ac.uk/arrayexpress), were interrogated for the expression of TWIST1, TWIST2, SNAI1, SNAI2, E-cadherin, N-Cadherin and Vimentin. The probe-level raw data (.CEL files from Affymetrix chips) were imported into BRB-ArrayTools 3.7 software package (http://linus.nci.nih.gov/BRB-ArrayTools.html) that allowed to compute probeset summaries using the gcrma method, to apply a log2 transformation to the signal intensities and to normalize arrays using the median normalization over entire array method. Gene expression data were then extracted using the dedicated plug-in utility and analyzed as described in the statistical analysis section.

When more than one probe set matched a given gene, the one with the highest variance was selected. Pearson's correlation coefficient was used to assess the correlation between gene expression levels.

Only 1 out of the 11 datasets available included TWIST2 probe sets and provided survival data [28]. This dataset (Dataset XI in Supplementary Table 1) was then interrogated to assess the impact of TWIST2 expression on HNSCC aggressiveness by Kaplan-Meier method.

## Supplementary Figures and Tables

Supplementary Figures

Supplementary Tables
