# Supplementary material for: Overexpression of TWIST2 correlates with poor prognosis in Head and Neck Squamous Cell Carcinomas
Source: Oncotarget. 2011 Dec 22;2(12):1165–75. doi: 10.18632/oncotarget.390 (PMC3282075; doi:10.18632/oncotarget.390)
Supplement: Supplementary Figures [file oncotarget-02-1165-s001.pdf]

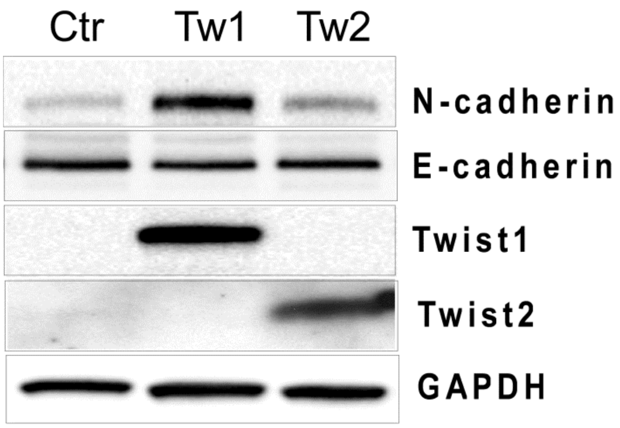

Supplementary Figure 1. Ectopic Twist2 expression fails to induce gain of mesenchymal features. Cells were transfected with TWIST1 (Tw1), TWIST2 (Tw2) or control empty vector (Ctr). Seventy-two hours after transfection, cells were harvested and analyzed for protein expression by Western Blot. GAPDH was used for normalization. Ectopic expression of TWIST2 failed to affect N-cadherin and E-cadherin expression.

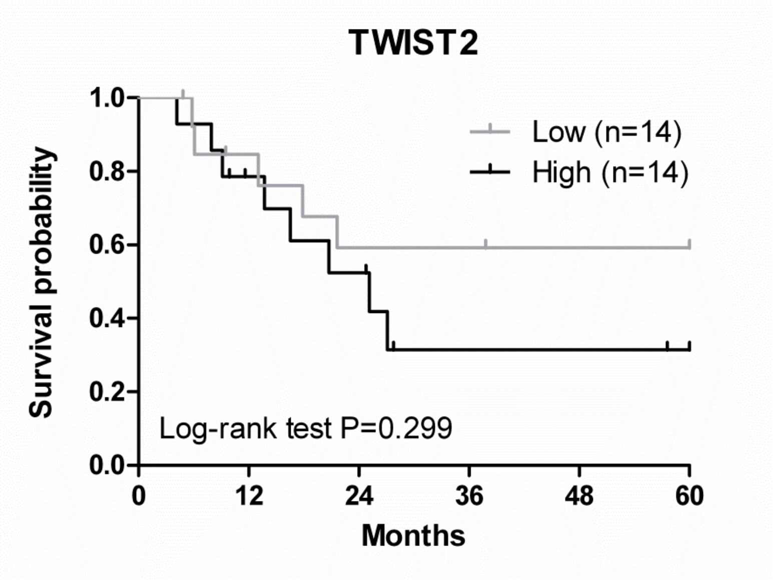

Supplementary Figure 2. Kaplan-Meier estimates of recurrence-free survival according to TWIST2 expression in the HNSCC dataset by Chung and coll. (2006) (Dataset XI in Supplementary table 1) [28]. Tumors were divided into high expressors (black line) and low expressors (grey line) using the median value of TWIST2 expression as a cutoff.
