# Supplementary material for: Overexpression of TWIST2 correlates with poor prognosis in Head and Neck Squamous Cell Carcinomas
Source: Oncotarget. 2011 Dec 22;2(12):1165–75. doi: 10.18632/oncotarget.390 (PMC3282075; doi:10.18632/oncotarget.390)
Supplement: Supplementary Tables [file oncotarget-02-1165-s002.pdf]

SUPPLEMENTARY TABLE 1. Pearson's correlation in HNSCC microarray datasets

Dataset				HNSCC Samples		
**Code**	**Database**	**Reference**	**Platform**	**Pharynx Oral cavity**	**Larynx**	**Total**
**I**	Oncomine	Ye et al.<br>BMC Genomics 2008	Affymetrix<br>U133 Plus 2.0	26		26
**II**	Oncomine	Slebos et al.<br>Clin Cancer Res 2006	Affymetrix<br>U133 Plus 2.0	27	9	36
**III**	Oncomine	O`Donnell et al.<br>Oncogene 2005	Affymetrix<br>U133A	26	1	27
**IV**	Oncomine	Ginos et al.<br>Cancer Res 2004	Affymetrix<br>U133A	26	15	41
**V**	Oncomine	Toruner et al.<br>Cancer Genet Cytogenet 2004	Affymetrix<br>U133A	16		16
**VI**	Oncomine	Kuriakose et al.<br>Cell Mol Life Sci 2004	Affymetrix<br>U95A-Av2	18	4	22
**VII**	Oncomine	Talbot et al.<br>Cancer Res 2005	Affymetrix<br>U95A-Av2	31		31
**VIII**	Oncomine	Cromer et al.<br>Oncogene 2004	Affymetrix<br>U95A-Av2	31		31
**IX**	Oncomine	Hensen et al.<br>BMC Cancer 2008	Affymetrix<br>Human Genome Focus	14	8	22
**X**	Oncomine	Roepman et al.<br>Nat Genet 2005	Human Array-Ready<br>Oligo set (v 2.0)	Not specified	Not specified	109
**XI**	EBI Array Express	Chung et al.<br>Cancer Res 2006	Affymetrix<br>U133_X3P	23	5	28

Dataset I (Ye et al. 2008)

Gene	E	N	V	S1	S2	T1	T2
**E**	1	-0.6	0.1	-0.3	0.1	-0.4	0.3
**N**		1	0.3	0	0.5	0.6	-0.3
**V**			1	-0.5	0.2	0.5	0.2
**S1**				1	-0.4	0	-0.4
**S2**					1	0.4	0.2
**T1**						1	0.2
**T2**							1

Dataset II (Slebos et al. 2006)

Gene	E	N	V	S1	S2	T1	T2
**E**	1	-0.2	-0.5	-0.2	-0.2	-0.5	-0.5
**N**		1	0.7	0.2	0.4	0.6	0.4
**V**			1	0.5	0.5	0.7	0.6
**S1**				1	0.1	0.2	0.5
**S2**					1	0.6	0.4
**T1**						1	0.7
**T2**							1

Dataset III (O'Donnell et al. 2005)

Gene	E	N	V	S1	S2	T1	T2
**E**	1	-0.1	-0.3	0	-0.2	-0.3	na
**N**		1	0.4	-0.1	0	0.3	na
**V**			1	0.3	0.1	0.4	na
**S1**				1	-0.1	0	na
**S2**					1	0.3	na
**T1**						1	na
**T2**							na

Dataset IV (Ginos et al. 2004)

Gene	E	N	V	S1	S2	T1	T2
**E**	1	0.3	-0.6	-0.1	0.5	-0.3	na
**N**		1	0.3	0.2	0.1	0.5	na
**V**			1	0.2	-0.2	0.5	na
**S1**				1	-0.2	0.2	na
**S2**					1	0.4	na
**T1**						1	na
**T2**							na

Dataset V (Toruner et al. 2004)

Gene	E	N	V	S1	S2	T1	T2
**E**	1	-0.8	-0.6	na	-0.4	-0.8	na
**N**		1	0.7	na	0.1	0.8	na
**V**			1	na	0.3	0.8	na
**S1**					na	na	na
**S2**					1	0.4	na
**T1**						1	na
**T2**							na

Dataset VI (Kuriakose et al. 2004)

Gene	E	N	V	S1	S2	T1	T2
**E**	1	0.1	-0.4	na	0.3	-0.3	na
**N**		1	0.3	na	0.4	0.5	na
**V**			1	na	0.2	0.7	na
**S1**					na	na	na
**S2**					1	0.4	na
**T1**						1	na
**T2**							na

Dataset VII (Talbot et al. 2005)

Gene	E	N	V	S1	S2	T1	T2
**E**	1	-0.5	-0.6	na	0	-0.5	na
**N**		1	0.4	na	0.2	0.3	na
**V**			1	na	0.3	0.6	na
**S1**					na	na	na
**S2**					1	0.3	na
**T1**						1	na
**T2**							na

Dataset VIII (Cromer et al. 2004)

Gene	E	N	V	S1	S2	T1	T2
**E**	1	-0.1	-0.1	na	0.1	0	na
**N**		1	0	na	0.2	0.1	na
**V**			1	na	0.1	0.4	na
**S1**					na	na	na
**S2**					1	0.4	na
**T1**						1	na
**T2**							na

Dataset IX (Hensen et al. 2008)

Gene	E	N	V	S1	S2	T1	T2
**E**	1	-0.5	0	-0.2	0	-0.1	na
**N**		1	0.2	0.2	0.2	0.1	na
**V**			1	0.2	0.5	0.6	na
**S1**				1	0.1	0.4	na
**S2**					1	0.5	na
**T1**						1	na
**T2**							na

Dataset X (Roepman et al. 2005)

Gene	E	N	V	S1	S2	T1	T2
**E**	1	-0.4	-0.4	0	-0.1	0.1	-0.1
**N**		1	0.3	-0.1	0.2	0	0
**V**			1	0.1	0.4	-0.1	0.1
**S1**				1	0.2	0.1	0.1
**S2**					1	0	0.2
**T1**						1	0
**T2**							1

Dataset XI (Chung et al. 2006)

Gene	E	N	V	S1	S2	T1	T2
**E**	1	0.5	-0.1	-0.5	0.6	0.7	0.6
**N**		1	0	-0.3	0.7	0.6	0.6
**V**			1	0.4	0.1	-0.1	-0.2
**S1**				1	-0.4	-0.3	-0.4
**S2**					1	0.6	0.4
**T1**						1	0.4
**T2**							1

Legend:

E, E-cadherin
N, N-cadherin
V, Vimentin
S1, SNAI1
S2, SNAI2
T1, TWIST1
T2, TWIST2

Supplementary Table 2. Correlation of the expression levels of EMT-related genes with pathological nodal status and tumor grading

	Nodal status				Tumor grading*			
	N0		N+		Well/Moderately differentiated		Poorly differentiated	
	n	(%)	n	(%)	n	(%)	n	(%)
**TWIST1**								
Low†	9	(45.0)	25	(52.1)	20	(51.3)	15	(50.0)
High†	11	(55.0)	23	(47.9)	19	(48.7)	15	(50.0)
Fisher test			p=0.791				p=1.000	
**TWIST2**								
Low	8	(40.0)	26	(54.2)	25	(64.1)	10	(33.3)
High	12	(60.0)	22	(45.8)	14	(35.9)	20	(66.7)
Fisher test			p=0.425				p=0.016	
**SNAI1**								
Low	8	(40.0)	27	(56.3)	21	(53.9)	14	(46.7)
High	12	(60.0)	21	(43.8)	18	(46.2)	16	(53.3)
Fisher test			p=0.290				p=0.631	
**SNAI2**								
Low	9	(47.4)	25	(51.1)	20	(52.6)	14	(48.3)
High	10	(52.6)	22	(48.9)	18	(47.4)	15	(51.7)
Fisher test			p=0.787				p=0.807	
**E-Cadherin**								
Low	9	(45.0)	25	(53.2)	17	(43.6)	17	(58.6)
High	11	(55.0)	22	(46.8)	22	(56.4)	12	(41.4)
Fisher test			p=0.600				p=0.327	
**N-Cadherin**								
Low	8	(44.4)	23	(52.3)	17	(50.0)	15	(51.7)
High	10	(55.6)	21	(47.7)	17	(50.0)	14	(48.3)
Fisher test			p=0.780				p=1.000	
**Vimentin**								
Low	9	(45.0)	24	(52.2)	20	(52.6)	14	(48.3)
High	11	(55.0)	22	(47.8)	18	(47.4)	15	(51.7)
Fisher test			p=0.789				p=0.807	
**N-Caderin/Vimentin**								
Other	11	(61.1)	31	(70.5)	22	(64.7)	21	(72.4)
High/High	7	(38.9)	13	(29.6)	12	(35.3)	8	(27.6)
Fisher test			p=0.554				p=0.593	

*Some figures do not add up to total because of missing values.
†High and Low indicate the samples with expression levels above and equal/below the median value.

Supplementary Table 3. Kaplan-Meier estimates, hazard ratio of death (HR) and corresponding 95% confidence intervals (CI) according to patient age, tumor characteristics, and gene expression

Characteristic	n all	n per site	3-years survival probability	5-years survival probability	Log-rank test	HR (95% CI)§
**Age (years)**						
<60	41	5 L; 36 OP	51%	44%		1†
≥60	28	11 L; 17 OP	54%	46%	p=0.839	1.17 (0.60-2.28)
**Site**						
Oral cavity/Pharynx (OP)	53	53 OP	45%	37%		1†
Larynx (L)	16	16 L	75%	69%	p=0.030	0.35 (0.13-0.93)
**Nodal status**						
N0	20	10 L; 10 OP	80%	70%		1†
N+	48	5 L; 43 OP	40%	33%	p=0.004	2.59 (1.04-6.44)
**Grading**						
Well/Moderately differentiated	39	13 L; 26 OP	62%	56%		1†
Poorly differentiated	30	3 L, 27 OP	40%	30%	p=0.031	1.54 (0.88-2.70)
**Stage**						
I-II	9	3 L; 6 OP	89%	78%		1†
III-IV	60	13 L; 47 OP	47%	40%	p=0.043	3.80 (0.91-15.81)
**TWIST1**						
Low	35	8 L, 27 OP	46%	40%		1‡
High	34	8 L; 26 OP	59%	50%	p=0.277	0.70 (0.37-1.34)
**TWIST2**						
Low	35	9 L; 26 OP	60%	51%		1‡
High	34	7 L; 27 OP	44%	38%	p=0.160	1.57 (0.83-2.98)
**SNAI1**						
Low	35	8 L; 27 OP	60%	48%		1‡
High	34	8 L; 27 OP	44%	38%	p=0.535	1.25 (0.65-2.40)
**SNAI2**						
Low	34	7 L; 27 OP	50%	41%		1‡
High	33	8 L; 25 OP	55%	48%	p=0.344	0.75 (0.39-1.44)
**E-Cadherin**						
Low	34	5 L; 29 OP	47%	38%		1‡
High	34	11 L; 23 OP	56%	50%	p=0.604	0.86 (0.45-1.64)
**N-Cadherin**						
Low	32	6 L; 26 OP	56%	47%		1‡
High	31	8 L; 23 OP	52%	48%	p=0.870	0.98 (0.49-2.01)
**Vimentin**						
Low	34	8 L; 26 OP	53%	47%		1‡
High	33	7 L; 26 OP	55%	48%	p=0.609	0.86 (0.44-1.65)

§Estimated by means of Cox hazard and adjusted for age and cancer site.
†Reference category. Survival curves were truncated at 5 years of follow-up.
‡Reference category. High and Low indicate the samples with expression levels over and equal/below the median value. The analysis of SNAI2, E-Cadherin, N-Cadherin and Vimentin was performed on 67, 68, 63, 67 cases, respectively, due to sample shortage.
